# Multicultural Implementation-Experiences of Peer Support Workers in MH Services: Qualitative Findings of UPSIDES Innovative Intervention—An International Multi-Site Project

**DOI:** 10.1007/s10926-025-10320-4

**Published:** 2025-11-03

**Authors:** G. S. Moran, Y. Goldfarb, I. Adler Ben-Dor, S. Hadas-Grundman, J. Kalha, D. Baillie, E. Kwebiiha, C. Mahlke, M. Ramesh, M. Slade, M. Haun, S. Krumm

**Affiliations:** 1 Department of Social Work, Ben Gurion University of the Negev, Beer Sheva, Israel; 2 School East London NHS Foundation Trust, London, UK; 3 Centre for Mental Health Law and Policy, Indian Law Society, Pune, India; 4 East London NHS Foundation Trust, London, UK; 5 Butabika National Referral Hospital, Kampala, Uganda; 6 Department of Psychiatry and Psychotherapy, University Medical Center Hamburg-Eppendorf, Hamburg, Germany; 7 Ifakara Health Institute, Dar es Salaam, Tanzania; 8 School of Health Sciences, Institute of Mental Health, University of Nottingham, Nottingham, UK; 9 Department of Psychiatry II, Ulm University, Ulm, Germany

**Keywords:** Peer providers, Knowledge from experience, Multicultural mental health, System integration, Occupational development

## Abstract

**Purpose:**

Mental health peer support is growing as an essential recovery-oriented occupation that can help alleviate the burden of mental health (MH) conditions. Despite its international growth, there is lack of cross-cultural knowledge about peer support workers’ (PSW) implementation experiences. This study explored PSWs’ implementation experiences of Using Peer Support In Developing Empowering Mental Health Services (UPSIDES) innovative intervention—a multi-country project designed to empower and scale-up peer support in low-, middle- and high-income countries (LMIC & HIC).

**Method:**

Nine focus groups totaling 38 PSWs were conducted at six study sites: Ulm and Hamburg (Germany), Kampala (Uganda), Dar es Salaam (Tanzania), Be’er Sheva (Israel), and Pune (India). Transcripts were analyzed using qualitative analysis and MAXQDA software.

**Results:**

Four domains were identified: (i) *PSWs’ experiences of recovery*; (ii) *PSWs’ experiences of vocational development*; (iii) *PSWs’ experiences with staff  and work-role in MH systems*;and  (iv) *PSWs’ strive to influence broader circles*. In low-and-middle-income countries specifically, PSWs experienced gains for illness management, access to care, employability and financial standing. They also engaged in active interactions with stakeholders in the community (e.g., relatives, police, etc.) promoting social-educative and anti-stigma influences. In high-income countries, experiences related to self-disclosure and peer-vocational development issues. Across sites PSWs’ expressed challenges and needs related to lack of role clarity, relations with MH staff and integration into services.

**Conclusions:**

The findings reveal the potential of mental health peer support workers (MH PSWs) to aid in mental health recovery and systemic change. However, implementation experiences vary across cultures, highlighting the need to further develop the PSW role and integrate it into mental health systems across different sites.

Trial Registration: ISRCTN26008944.

**Supplementary Information:**

The online version contains supplementary material available at 10.1007/s10926-025-10320-4.

## Introduction

Many individuals in need of mental health services receive inadequate care in high-income countries. This is even more pronounced in low- and middle-income countries, which have a massive disparity between the populations’ mental health needs and the availability of trained mental health human resources [1, 2]. One way to address this gap is by developing new models of treatment that engage lay workers [2, 3]. Peer support for mental health problems has expanded globally in the past few decades and have been suggested as one way to address this growing public health need [4, 5]. At the same time becoming a peer support worker provides a means of occupation to persons with severe mental health conditions.

## Mental Health Peer Support—Unique Characteristics and Challenges

Peer support in mental health (MH) is a unique workforce in the landscape of mental health professions. Peer support interventions align with the conceptual model of recovery defined as “a way of living a satisfying, hopeful, and contributing life even within the limitations caused by illness” (p.527) [6, 7]. This human centered approach (as opposed to an illness focused approach) is also known as “personal recovery” (contrasted with "clinical recovery") [8]. Recovery is a complex notion involving both personal and clinical aspects [8–10]: Personal recovery addresses the reclaiming of a meaningful life, and self-directed process of regaining a valued role and citizen in society [8], while clinical recovery relates to remission of symptoms [11].

Drawing on their own shared experiences, mental health peer support workers (PSWs) role-model that recovery is possible and build an empathetic camaraderie with service users.  They derive their credibility as experts-by-experience for having “been there”—having experienced mental health symptoms and having learned how to navigate the MH system. PSWs provide practical and emotional support, positive self-disclosure, promote hope, empowerment, self-efficacy, and an expanded social network [12–14].

PSWs are an integral part of recovery and citizenship approaches, by aiming to promote the dignity, rights and full participation of people with severe mental health conditions in society. They aim to reduce stigma, promote empowerment, and increase a sense of belonging for those in recovery [1, 8, 15]. While PSWs’ integration can elicit systemic and personal challenges there is growing recognition of their potential contribution and continued efforts of implementation in MH care services [11, 16–18].

Mental health peer support has been increasingly implemented and studied in high-income countries such as USA [19, 20], UK [9, 21–23], Europe [24, 25], Israel [26, 27], Australia and New Zealand [28–31]. It has been studied to some degree in countries in Africa such as Uganda [32, 33] and South Africa [16], India [34, 35], China and Hong Kong [36, 37], as well as in South American countries, such as Brazil [15]. Yet, overall, mental health peer support is understudied in low- and middle-income countries. Moreover, there is considerably less research that examines peer support within a single intervention program across low, middle and high-income countries [13, 38]. By conducting research across diverse countries, one can ensure that peer support programs are inclusive, effective, and adaptable to different contexts, ultimately improving mental health outcomes for individuals globally.

## Research Goal and Context of the Current Study

This study is part of an international science implementation project: UPSIDES (Using Peer Support In Developing Empowering Mental Health Services, www.upsides.org)—a multi-center international science implementation project. UPSIDES is a five-year, six-country study which set out to replicate and scale-up peer support interventions for people with severe mental illness (SMI), generating evidence for sustainable best practices in high-middle and low-income countries [39, 40]. The program includes low-income (Kampala, Dar es Salaam), lower-middle (Pune) and high-income sites (Ulm, Hamburg, Beer Sheva). This pragmatic multi-center parallel-group wait-list randomized controlled trial took place at the six study sites, assessing social inclusion (primary), empowerment and hope outcomes as well as a host of additional clinical aspects [39]. Other  studies from this project involved a manual development using theory of change for the intervention, situational analysis in each site for peer support implementation, examination of MH workers and stakeholders’ experiences, and more, providing rich and nuanced understanding of process and implementation across multiple stakeholders [41–43]. [42, 44 Implementation was supported by *an organizational package* which includes educational activities for staff, and on-going supervision for PSWs. The project was also conducted during the Covid pandemic, gaining insights into its value in such crises times (for more description and publications from the international project see www.upsides.org; 46).

 This study makes a distinct contribution to the peer support evidence base by focusing on PSWs' experiences in low, Middle- and high- income countries with training and implementing the innovative UPSIDES intervention. [39, 40]. Such knowledge is important for two reasons: (a) to learn about an internationally developed peer support intervention’s implementation in both developed sites and more traditional cultures and poorer countries which face a dire public health challenge of workforce shortage, and (b) to examine whether the implementation of UPSIDES intervention succeeds in sharing the same vision of peer values (reciprocity, role modeling), recovery values and principles (e.g., person centered, self-determination). Knowledge from this study is expected to support  peer interventions to fulfill their contribution across diverse organizational, economic, and cultural contexts [13, 34, 47, 48].

## Method

### UPSIDES Peer Support Intervention and Context of the Study

UPSIDES intervention was developed and piloted based on state-of-the art knowledge on mental health peer interventions and local knowledge from each participating site peers and stakeholders. The UPSIDES intervention adheres to core peer principles (e.g., sharing lived experience, mutuality) and includes 12 standardized modules which can be adapted simultaneously for different contexts. UPSIDES can be delivered either one-on-one or in a group format, in medical or community mental health services [42, 44, 45]. It is delivered for up to 6 months with a minimum of three meetings with peer service recipients. PSWs were trained to show empathy, authenticity and respect, and mutually give and take. Sessions focused on working out solutions together, empowerment and helping participants grow.

PSWs were further trained to inspire hope in others, show possibilities and opportunities and promote autonomy. Inclusion of different perspectives, sharing knowledge and supporting service users to participate in society were also encouraged [44]. Senior peer trainers were invited from each local site for a 5-day training in Tanzania, and then continued to train and deliver the UPSIDES intervention on in their own local sites. The intervention was flexibly designed in order to allow adaptation to local needs and differing mental health settings [39, 40, 42, 44]. The study sites differed in terms of urban versus rural setting, service provision (inpatient vs outpatient, community service) and the extent of their prior experience with peer support (see supplemental file 1).

### Focus Group Participants

Focus groups with PSWs took place at study sites in Pune (India), Be’er Sheva (Israel), Dar es Salaam (Tanzania), Hamburg (Germany), Butabika (Uganda), Ulm/ Guenzburg (Germany) and Hamburg (Germany). PSWs who underwent UPSIDES training and delivered the intervention to service users were invited to participate in focus groups following the completion of the intervention. In total 38 participants took part in nine focus group discussions (FGDs). Twenty-four participants were female, 13 participants male and one participant defined their gender as fluid. Participants’ age ranged from 25 to71, with an average age of 43 (see Table 1).

**Table 1:** Characteristics of focus group across study sites

Study Site	Ulm/ Guenzburg Germany (Ulm)	Butabika, Uganda (BU)		Pune, India (PU)	Be’er Sheva, Israel (BGU)		Hamburg, Germany (UKE)	Dar es Salaam, Tanzania (DS)	
N (Gender)	6 (*f* = 3, *m* = 3)	3(f = 0, *m* = 3)	5(*f* = 5, *m* = 0)	3(*f* = 2, *m* = 1)	6(*f* = 5, *m* = 1)	4(*f* = 2, *m* = 2)	5(*f* = 3, *m* = 1, *d* = 1)	4 (*f* = 4, *m* = 0)	2(*f* = 0, *m* = 2)
Mean age in years (range)	51 (37–62)	41.3(32–47)	36.8(27–46)	45(40–50)	41(32–50)	41(33–57)	54.4(40–71)	33.33(27–38)	28(31 − 25)
Date	06/09 2021	13/08 2022	13/08 2022	18/04 2022	28/07 2021	28/07 2021	20/10 2021	16/12 2021	21/01 2022
Duration	100 min	130 min	105 min	32 min	90 min	90 min	120 min	135 min	68 min

### Procedure and Data Collection

PSWs who performed the UPSIDES intervention were approached by the research teams and invited to participate in focus groups after completing the intervention delivery. FGDs were conducted between July 2021 and April 2022, about 18 months after the UPSIDES intervention has started. Participants were provided with study information (oral and written forms) and signed an informed consent form (approved by ethics committee—see Ethic statement for more information). Data were collected using a semi-structured topic guide. The topic guide was developed collaboratively by research workers from the study sites in Israel and Germany (Ulm). The final topic guide included the following four topics: (1) PSWs Experiences in training and working in UPSIDES; (2) UPSIDES effects on service users; (3) Organizational issues (contextual factors, barriers, and facilitators); and (4) effects of COVID-19. See supplementary file 2 for the full topic guide.

FGDs were conducted in the local language or English (where participants had sufficient fluency) and were audio-recorded. Each FGD was facilitated by a moderator and an assistant, who had prior training or experience in qualitative data collection. At the end of the FGD a short questionnaire was given to participants to gather basic demographic information including participants’ gender, age, and professional background. After each session, the moderator and assistant wrote field notes capturing their impressions of the dynamics of the interactions and insights from the discussion related to the main topics and research questions.

### Transcription and Translation

Study materials including topic guides were forward translated from English to the local language by a bilingual speaker at each study site. For increased feasibility, adaptations were made to some questions by rephrasing them without altering their core meaning. Audio files were transcribed verbatim in the language used in the FGD sessions. Personal information in the transcripts was replaced with participant numbers. Transcripts were then translated to English by a bilingual speaker. Two researchers as part of the UPSIDES translation team checked all final translated transcripts to ensure comprehensibility for analysis.

### Analysis

A qualitative study of PSWs’ experiences of the intervention offers a deeper insight into personal experiences of PSWs, active ingredients, processes and manifestations of implementing UPSIDES intervention as well as barriers. COREQ guidelines [49] are available in supplementary file 3.

The transcripts of FG were analyzed with MAXQDA 24 software, employing thematic analysis   helped to examine how themes manifest across different cultural contexts while maintaining a systematic approach to identifying commonalities and differences [50]. Analytic procedure followed these steps: (1) **familiarization and initial coding**—YG and IAB independently read and analyzed the same transcript, suggesting initial codes and themes inductively from the data; (2) **searching for themes**—then codes were grouped into broader themes that capture main themes guided by the research questions. (3) **refining and defining themes**—together with GSM themes would be reviewed and discussed for coherence. If need be re-conceptualized, merged, and/or eliminated, resulting with the final definition and description of themes. (4) **cross-site validation**: themes were validated with research workers at each site to confirm data alignment.

This strategy provides an important check on potential selective perception and blind interpretive bias too [51]. To ensure trustworthiness of the data and cultural understanding analyses were shared and reviewed with local teams to verify understanding. In addition, online meetings were arranged with each site’s local research teams to assure understanding of contextual cultural and organizational context (see also supplementary file 1).

Each FG was considered and analyzed as a separate unit of analysis, therefore quotes refer to the site and location of the FG and the position of paragraph in the sequence of the group discussion.

## Findings

The study focus involves mainly outcomes from the first topic in the topic guide regarding the experiences of peer support workers which were most insightful in regard to peer support implementation in a cross-cultural context. Other topics were not included in this analysis because they had less innovative value regarding our specific study questions (e.g., more nuanced findings about benefits for recovery). Through the analytic process, PSWs’ implementation experiences were identified under four overarching themes (i) PSWs’ experiences of recovery, (ii) PSWs’ experiences of vocational development, (iii) PSWs’ experiences with staff and their work-role in MH systems, and (iv) PSWs’ strives for broader influence—among family, community and the general public. Under each theme, two subthemes have been identified. Most of the findings pertain to PSWs’ shared experience across sites. In fewer instances some site-specific issues were also identified and related to low-, middle- and high-income countries (LMIC & HIC) sites (see Table 2).

**Table 2:** Findings

Themes, subthemes and additional quotes		
Theme		Example quotes
*i. PSWs’ experiences of recovery*		
Subthemes	1.1 Promoting personal recovery through UPSIDES training and intervention	“It has increased confidence as well and we felt better knowing that we could help someone too. We are not just someone who is struggling with mental illness and this gave a lot of strength and we kept getting solutions “ (Pune, 198) This program teaches you to give…you see success, how it relates to me, it’s uplifting for me… When you give, you receive.. You suddenly feel, wow, I saved someone… When you give- you receive. Giving is the greatest receiving, Rabbi Dessler said that. And it’s true, in my opinion. I do believe in this saying.” (Beer Sheva, Group 1, 379)
	1.2 Enhanced illness management in LMIC	“I got to understand about my mental illness even more… whenever the triggers are increased, I am able to recognize them.” (Pune, 196)
*ii. PSWs’ experiences of vocational development*		
Subtheme	2.1 Relational skills and self-disclosure practices of lived experiences	“[following UPSIDES training. G.M.].. It's becoming increasingly easier for me to talk about myself and my sensitivities, something that used to be very shameful for me.” (Ulm) “before I received training, I was able to share.... information that I was not supposed to share but I was sharing. So, the circle of safety has enabled me to understand that there is some information which we are supposed to share with people and other information that you need to keep to yourself…so the way we were taught on how to solve challenges has been able to help me”. (Dar es Salaam)
	2.2 Vocational and financial skills in LMIC	“It [UPSIDES] has taught me about how to spend my money well and also help people, also the proper planning of the use of money… through this project I have been able to save some money… I have been able to do many things through the salary I earn from UPSIDES because my mind has focused on projects that can bring profit”. (Dar es Salaam)
*iii. PSWs’ experiences with staff and their work-role in MH systems*		
Subtheme	3.1 Relations with mental health staff	“I think if the staff were more involved then these connections would have produced something in the level of the service and the organization, there were some added value… generally speaking, to take interest…. to be with us….“(Dar es Salaam)
	3.2 Challenges in role and system integration	“it’s very significant- without the support of the director it would be very hard, … in my opinion even that workers with knowledge from experience [i.e., PSWs] would be in a position which is more defined… there should be a conversation with the staff all the time and they should be involved with the language we are trying to bring….with the ability to see the person as a person and not as a rehabilitator or coper or tenant or…. only as a human being and it’s something that can be done with the directors and the staff members..” (Beer Sheva]
*iv. PSWs’ strives to influence broader circles—family, community and the general public*		
	4.1 Countering stigma and raising awareness	“I think we could also use… a television station, which also makes short clips… and I think that these are also viewers where someone might think, yes, maybe this concerns me myself, I can use it, or I know who might need it" (Hamburg)
	4.2 Influence on community and other stakeholders in LMICs	“Resettling the patients who have been discharged has also helped their families so much.” (Kampala) “There was this one service user, she used to come with her husband. The husband got really furious at me.. he used to beat her. I gave her that paper [list of welfare resources] the husband did not realize it.” (Pune)

### (i) PSWs’ Experiences of Recovery

Across sites, many PSWs shared that by training and implementing the UPSIDES intervention they experienced multiple subjective and objective benefits to their own recovery processes. This included experiencing enhanced self-knowledge and empowerment to one’s own recovery: by becoming peer supporters, participants’ motivation to safeguard their own MH increased by wanting to be well for those they serve. Other PSWs’ shared they experienced wellbeing through the PSW role by gaining a sense of confidence, self-esteem and self-efficacy. Additionally, many PSWs in LMIC reported gaining tangible benefits related to their PSWs’ role, such as managing one’s own mental health and gaining direct access to care through UPSIDES training and services. These themes are described with demonstrative quotes next.

#### Promoting Personal Recovery Through UPSIDES Training and Intervention

Gaining personal benefits to one’s own recovery were described as early as during training:

“This topic of the tree of life, it has taught me about my history and where I am currently and where I am going to. Before receiving training, it reached a point where I had lost hope" (Dar es Salaam, group 1 female, 34).

In this quote the PSW referred to how an exercise in UPSIDES training retrieved back their sense of hope. Another quote demonstrates enhanced motivation for self-care in order to be there for others:

“It [UPSIDES] also helped me work on my own recovery knowing that I was a role model so definitely knew that if I stop my medication or do something that could trigger a relapse…, then my peers [service users] would be discouraged” (Kampala, group 1, female, 32)

Some PSWs specifically referred to sensing empowerment and enhanced self-esteem by helping others: “When you give, you receive. You suddenly feel, wow, I saved someone” (Beer Sheva, group 1, 379). As well as by social comparison:

“I am reminded of my story and think, ah, wow. I've come a long way. He will do it too, she will do it too and then I can be a bit proud of myself, how far I have come” (Ulm, 89).

#### Enhancing Illness Management in LMICs

Many PSWs in LMIC additionally described better management of their illness (becoming self-aware, learning to identify triggers and symptoms and how to take care of them), and improved access to mental health care (direct connection with professionals, access to medication). For example, a PSW from India described their UPSIDES training experience as follows:

“It also helped me with my current mental illness. Like when during the training she [trainer, AU.I.] asked us a few questions like ‘what do you do when you are triggered? How do you balance between your mental health and physical health?’ And we were also given a form to maintain it personally. And I do maintain that personally at home. And that has really helped me a lot” (Pune, 34).

In addition, many PSWs gained timely access to MH services and supports for their own needs. Accordingly, a PSW from Dar es Salaam (Tanzania) said: “it has become easy now for me to access services, for example if I face a problem, I can contact madam (OT) and she gives me support easily.” (Dar es Salaam, group 2, male, 32). Another said: “The community team was helpful to us all PSWs because they gave us priority. They made sure we had our medications because there would be a time when the drugs are scarce “ (Kampala, group 1, female, 76).

### PSWs’ Experiences of Vocational Development

Across sites, most PSWs experienced skill development of various types which related to their work as peers. Relational skills, such as sharing personal lived experiences, were experienced by most PSWs across all sites. Certain skills and vocational development were more prominently experienced in different HIC of LIC sites.

In HIC sites (Hamburg, Ulm & Beer Sheva) PSWs reported experiences related to development of a peer identity work-role as well as personal processes related to their professional development. In LIC sites, many PSWs reported experiencing development of skills that expanded to broader life domains, involving relational and vocational development as well as gaining a financial standing. These themes are detailed with quotes next.

#### Developing Relational Skills and Self-Disclosure Practice of Lived Experience

PSWs improved their interpersonal skills as well as developed ways of sharing their lived experiences with service users and others. The next 2 quotes demonstrate growth in interpersonal skills which contributed to their personal and vocational lives:

“...I was so scared to speak in front of other people, and I couldn’t establish a relationship, but the UPSIDES project has changed me… I have met people face to face and I have been able to talk to them, it has changed me, and I can change my career to become an educator” (Dar es Salaam, group 1, female, 103)

“I also got some skills that have helped me in my personal relationship for example training on conflict resolution has been very helpful. I also got skills in speaking; basically, being able to talk to someone and listen. And also, self-disclosure…to build rapport“ (Kampala, group 1, female, 52)

The following quotes portray PSWs positive experiences and peer identity development that occurred by receiving supervision and practicing self-disclosure of lived experiences: “I didn’t really know at the beginning how to deal with closeness and distance… with difficult situations… and that’s when I found supervision… totally helpful” (Hamburg, 15).

“UPSIDES gave some sort of guidance to self-disclose myself [e.g., having lived experience] in a way I really wanted to and couldn’t [before the training], something in the training helped me formulate this sort of identity of being a worker with knowledge [from experience]” (Beer Sheva, group 2, 18).

#### Developing Vocational and Financial Skills in LIC

Many PSWs in LIC reported on vocational development, improved interpersonal relations, employability and financial independence. For example, a PSW shared: “I had a mental health challenge and was fired from work. So, the project helped me to have another employment opportunity” (Dar es Salaam, group 1, female, 103). Some PSWs learned to better plan and use their capital, leading to further constructive monetary actions such as paying for school fees or starting a business. A peer from Uganda said:

“In the training the facilitators told us to think about some businesses to do with the little money at our disposal... I had little capital, I started doing liquid soap in my house and I sell it in my area and am able to attain needs for the family through such a small business” (Kampala, group 2, male, 65).

### PSWs’ Experiences with Staff and Their Work-Role in MH Systems

The relationships and work-role of PSWs in the mental health settings where they implemented UPSIDES peer intervention were brought up by most PSWs in the study. Many experienced positive relations with mental health staff (although not all). Such positive relations were emphasized as meaningful to their peer roles and recovery processes across sites. In addition, across sites, PSWs addressed issues and challenges related to having a PSW role within mental health services. These themes are demonstrated in quotes next.

#### Relations Between PSWs and MH Staff

Across all sites, PSWs appreciated when having experienced positive relations with staff. For example, PSWs’ relations with staff in Uganda, were mainly described as welcoming and inclusive:

“…the fact that they would not segregate against us…, at the ward they were really receptive, initially they would call out the patients for me.. I would reach there, and they would say; ‘so you also want us to go call for you patients…go there and look for them’ so it was like…, it would make me feel like I am one of them”. (Kampala, group 1, female, 76).

Another PSW from Beer Sheva also took to heart staff’s positive approach to PSWs’ presence in their service: “they accepted us with a lot of love… the staff was also waiting for us and appreciated… they had a very hard time when we canceled” (Beer Sheva, group 1, 777).

In LMIC, many PSWs described relationships with staff that involved staff taking care of PSWs: “They care about our wellness as peer support workers… they monitor our time for reviews…, they remind us to refill our medications…, they are like our mothers…, they care for us very well.” (Kampala, group 2, male, 106). In Pune PSWs similarly established supportive and health-supporting relationship with staff: “The staff here is very good and treat us well. When they scold us, we do feel bad, but they do treat us well. And because of that half of our illness is improved. I am happy” (Pune, 298).

Yet, in other instances, PSWs’ relations with mental health staff were described as distant or non-existent. In these instances, PSWs yearned for the relations to be closer. For example, one PSW had hoped that “staff were more involved…” and “generally speaking, to take interest… to be with us” (Beer Sheva, group 2, 312). Another PSW said: “They are always so busy on the ward, and we really have almost no contact with the staff of the hospital” (Ulm, 266). Finally, another PSW expressed disappointment because of lack of partnership with staff:

“On my part I think that the department here at the hospital has cooperated with us well or has received the project well but one of the challenges is that they had isolated themselves. They received us and received the project but the partnership to work as a team has been a bit of a problem.” (Kampala, group 2, male, 55).

#### Challenges in PSW Role Definitions and MH System Integration

Across sites some PSWs shared having experienced challenges in recognizing PSWs as a distinct provider-role, understanding its meaning and significance for all stakeholders involved: service users, PSWs and staff. PSWs stressed the importance of getting peer support work familiarized and enhancing awareness and support for peers in MH systems.

For example, in Pune, a PSW asked for “a dress code or ID card” (Pune, 245) so PSWs aren’t mistaken for service users: “Everyone thinks that we are also there to take medicines… if we are in a dress code, they will treat us normally. They will then understand our value” (Pune, 249). Another PSW from Tanzania complained they were approached with suspicion by service users:

“I think that there is some sort of difficulty when the PSW meets with the service user for the first time to provide peer support service and share their mental health challenges, you find that I am a newcomer to the service user, it becomes a little difficult for him to trust you” (Dar es Salaam, group 2, male, 16).

In Beer Sheva, a few PSWs faced the challenge of holding a dual role: they engaged in services in which they previously worked as personal assistants (not related to sharing of lived experience) and now engaged in the same services as PSWs: “I felt ambivalent because I wanted to be both a peer specialist and also their rehabilitation instructor, sort of a peer specialist who is also a rehabilitation instructor and that there will be continuity of it" (Beer Sheva, group 2, 132).

In addition, a few PSWs addressed the need to intentionally expose staff to the notion of peer support in MH services. For example, in Ulm, a PSW said:

“I think it definitely does good to introduce peer support in ward management meetings, at the nursing service management, at presentation events, so that it is simply anchored in the minds of the colleagues and in their awareness” (Ulm, 206).

Another PSW said:

“staff and management, I think… should be… there has to be sense of leadership, the director has to be into it, understand the meaning and the idea beyond it… it’s very significant without the support of the director it would be very hard” (Beer Sheva, group 2, 293).

### PSWs Strive to Influence Broader Circles—Family, Community and the General Public

Interestingly, across sites many PSWs reported on experiences and behaviors that extended beyond providing peer support services. These activities were sometimes linked to PSWs’ having been empowered in telling their personal story. PSWs reached out to broader circles and engaged in influential activities that promote awareness, educate about severe mental health conditions, enhance access to mental health care and counter stigma. These are described next with demonstrative quotes.

#### Raising Awareness and Countering Stigma

Some PSWs shared that they became empowered in telling their personal story, and developed motivation to initiate anti-stigma campaigns within and outside mental health field. For example:

“It [UPSIDES] helped me get confidence and come out on social medias and share videos about mental health, what helped me, and [how] I am using my medication. There are some who reached out and I spoke to them. It is not shameful to have mental illness that you cannot do anything in the community... [need to] pull the topic of mental illness out of the taboo corner” (Dar es Salaam, group 1, female, 174).

Another PSW working in a religious traditional service which was relatively stigmatic, discussed the lack of awareness within their mental health service and the role they see they have as PSWs to raise awareness, as follows:

“Awareness can only come by you carrying the message... we got there and declared and talked about it and carried some sort of message – ‘we are copers’ [i.e., coping with a mental illness rather than being ’mentally ill’..], and we bring experience-based knowledge, and we came to open up something ... and that is how it goes by word of mouth..” (Beer Sheva, group 2, 613).

In addition, some PSWs called for the continuation and expansion of the project, emphasizing the need for educational anti stigmatic actions. For example, a PSW from Pune called for PSWs to participate in the “International Olympic for disabled”. A PSW from Hamburg called to add video clips on TV related to mental health and peers. Another PSW suggested that PSWs reach out to the community as follows:

“We could visit schools, colleges, universities and the community could arrange for us to go to them and provide training full stop. For instance for me I had the first experience while at school so if this training will go to all these places, we are going to be able to reduce the problem and even if they get sick it won't be as much as when people do not have any knowledge this” (Dar es Salaam, group 1, female 152).

#### Reaching Out to Community Stakeholders in LMIC

Interestingly, different PSWs’ activities in LMICs went beyond the mental health system and targeted additional community stakeholders such as families, hospital staff, religious leaders, army, and police personnel as integral to their work. For example, PSWs shared that they reached out to relatives of service users and involved family members in their intervention. A PSW said they were “getting ways to go to their home and talk to them—both service users and their family for those who did not know how to handle service users.” (Dar es Salaam, group 1, female, 142). Another example involved a PSW who connected a service user with social-welfare services when identifying marital violence in Pune.

In Uganda encounters with the police were described extensively by a PSW as follows:

“.. I was supporting one of the soldiers that was mentally sick; they [the police] had beat him so much and were planning to take him to Butabika [Hospital]. So, I told the district police commander … ‘it can also happen to you…, to your wife and your children; you need to take it easy; I will handle this man” (Kampala, group 1, female, 59).

Following that incident and over time, the PSW continues to describe how the police changed their approach:

“they would call me to help out…, ‘come and help your friend; it’s only you that understands him’. whenever some of the officers would relapse on duty, they would call me; ‘come and help us’ and I would go…, and they would see me as their point of contact, whenever they wanted to get access to Butabika. So that was the beauty of it…, the effect of peer support." (Kampala, group 1, female, 59).

A PSW from Tanzania emphasized the risk of family and service users’ engagement with religious leaders and need to intervene: “instead of taking them [patients] to the hospital, they [family members] might take them to religious leaders… so the first priority would be that the organization in collaboration with UPSIDES to prepare programs that can help.” (Dar es Salaam, group 1, female, 155).

## Discussion

This study investigated peer support workers’ experiences of implementing UPSIDES innovative peer support intervention. This project was part of an international multi-center science implementation project designed to empower and up-scale peer support in middle-low- and high income countries [40]. This study is the first to examine PSWs' implementation experiences across diverse cultural and socio-economic contexts from their own perspectives, adding to the insights gained from other UPSIDES stakeholders [41]. .

UPSIDES implementation experiences were described by PSWs as transformative in terms of one’s being, thinking as well as interacting and operating in mental health systems and communities toward people with mental health conditions [52]. The multilevel impact described by PSWs involving both self, recipients of services, staff and outer community shows that UPSIDES was successfully implemented according to its goals as a recovery innovation with impact that expands to oneself, systems and communities [52]. Overall, the study indicates that within the flexible and culturally adaptive framework of UPSIDES, engaging in the roles of peer support workers (PSWs) fostered recovery for the PSWs themselves. It also empowered PSWs to carry out recovery-enhancing actions for service users and their environments. The findings are understood as relating to structural and cultural characteristics and interpreted in terms of site localities (MH systems vs. outer community).

Findings reveal both the shared common experiences of PSWs as well as specific processes and outcomes more characteristic in LMIC and HIC. More broadly, this study supports the notion that UPSIDES PSWs adhered to peer values and recovery orientation. Their training may be feasible in promoting global mental health and thus may be useful in response to the treatment gap in LMIC. Such knowledge can be useful to other implementation initiatives of peer support in diverse contexts with public and mental health needs [2, 4, 5].

### Benefits Experienced by PSWs Across Diverse Settings

Across sites, PSWs experienced benefits to their own recovery beginning with training and further on when engaging in peer support work roles. These benefits corroborate positive findings reported in other studies relating to the benefits to recovery of peer support workers [11, 53]. Like previous single country studies, here too, the engagement in UPSIDES peer support worker role involved enhancement in emotional well-being, social empowerment as well as employability [53–56]. Benefits for PSWs in implementation are often related to the therapist-helper principle [10]. Such motivation was identified in current PSWs’ personal experiences too—a motivation for self-care in order to be well and able to support others they will serve. This shows that peer roles enhance recovery irrespective of cultural background and pertain to common universal shared human experiences [8, 11].

The findings further y suggests that in LMIC PSWsoften experienced a positive upward spiral due to UPSIDES implementation. This occurred through mutual influences between one’s personal and clinical recovery: often, engaging in PS implementation enhanced one's motivation for illness management, which in turn enabled PSWs' vocational development and vice versa. Moreover, some PSWs pointed out the monetary reimbursement and the development of financial skills as critical for their own state and as a result also for successful implementation processes. PSWs’ engagement in peer roles contributed to their financial abilities and in some instances were transformative in securing their financial state through better financial handling, development of small businesses and employability. As noted above,  such implementation outcome further built motivation and confidence in one's PSWs’ work-role too.

Notably, LMIC involved PSWs in poorer financial status who may lack basic needs, including mental health and financial standing [43, 57]. The interface between poverty, lack of resources and mental health in LMIC is known to create a negative cycle that is hard to break [45, 59, 60]. These implementation outcomes speak to the role of the UPSIDES project in LMIC sites in developing vocational and financial capacities that support not only service users, but also solidifies PSWs’ own mental health, access to care and financial security in face of a scarce environment and lack of workforce.

### PSWs’ Relations with MH Services and Staff

PSWs in this study shared questions and issues regarding work-role definitions and integration in services. These reflect implementation challenges already identified in other studies [17, 45, 61] and  voiced by other stakeholders in the UPSIDES study [41]. Prominently, PSWs’ voiced their appreciation for close and supportive connections with MH staff contributing to optimal PSW-MH staff work collaborations. In LMIC, getting support for PSWs’ own mental illness  through staff were an integral part of the positive relations established.  In a mirror finding from the UPSIDES project MH workers emphasized the importance of trust and commitment in their relations with PSWs [62]. Together these findings emphasize the value of focusing on PSWs and MH workers relations in implementation processes and their unique value in LMIC for PSWs mental health well-being as well.

PSWs across sites were concerned with role related issues—self-presentation, self-disclosure, and role clarity and more, as well as the need for support from the systems for the development and solidifying of their role among staff. Specific needs varied according to context, cultural characteristics, and local development of peer roles. Overall the challenge of better integration in statutory mental health services was prominent across sites as described elsewhere [17, 41, 45, 46, 61]. The importance of organizational readiness and collaboration with MH services was noted also by PSWs as needed for the successful implementation of UPSIDES [43]. The current study’s multicultural PSWs perspective further supports the universality of these challenges.

Given these findings, we assume that near future implementation challenges cross-culturally will most likely continue to involve role clarity, leadership support, helping staff understand the values of recovery and contribution of peer support [17, 19, 58, 60, 63].

### From PSWs to Change Agents

Surprisingly, across sites PSWs shared how they extended their behaviors beyond the scope of UPSIDES intervention to larger circles in society. These intentional actsaddress lack of knowledge and awareness about severe mental health conditions, stigma and support in the general public. These implementation outcomes corroborate with the UPSIDES MH workers’ perspectives who described PSWs as bridges between hospital and the community [62] and other stakeholders in LMIC who identified  the essential role of integrating peer support into the local community [43]. The current study provides insight as to the nature of these interactions with the community. For example, PSWs interacted with stakeholders such as police, army and families to counter stigma, educate and increase their access to, and utilization  of mental health services. Some PSWs educated policemen and soldiers directly about illness management and made themselves available to them in times of need. These interventions were described as successful by PSWs, creating change in stakeholders’ approach to individuals with severe mental health conditions and learning to use PSWs when needed. Engaging stakeholders in the community is known to be helpful in reducing stigma, improving knowledge and attitudes toward mental health, and increasing efficient help-seeking [64, 65].

While PSWs in HIC did not expand activities outside the field of mental health as prominently as in LMIC sites, they did describe actions and intentions related to addressing stigma and awareness within the MH system. In addition, PSW in HIC were critically engaged in contemplating the development and consolidation of personal experiences in peer roles within the MH system. Similarly, the PSWs in LMIC countries in this study described using their personal stories with stakeholders, which made meaningful impact on stakeholders outside the MH system. By sharing one’s personal story PSWs transfer peers’ experience into mental health systems and thus contribute to their greater humanization [52, 66]. PSWs descriptions of these intentional acts in the community involve vicarious implementation outcomes that suggest UPSIDES implementation processes supported a deeper recovery innovation impact that goes beyond the mere intervention and service users [51].

Overall, the findings of the current study showed that PSWs in both HIC and LMIC sites developed a proactive “social change agent” approach. It demonstrated that PSWs, cross-culturally, can reduce stigma, promote empowerment, autonomy, activism, and advocate for others. In this way PSWs contribute to the recovery-citizenship approach [1, 8, 15].

The findings also highlight the importance of providing training, supervision and flexible supports for sustainability and adaptation of PSWs’ emerging roles in cross-cultural sites. As peer services are gaining recognition as a recovery-orientated approach and peer workforce is expanding in a global world because of shortage circumstances—knowledge from this study can inform implementation and career development of PSWs in diverse settings.

### Study Strengths and Limitations

The strengths of this study lie in its overall science implementation rigor [39, 40], development of the intervention with state-of-the-art knowledge and local stake holder inputs [45, 67, 68]. We believe the combination of the scholastic, locally sensitive approach and methodological rigor contributed to successful development and PSWs’ implementation experiences. The focus groups were conducted in the local language at each site pertaining to cultural soundness of data collection.

On the other hand, the researchers who analyzed the data were not directly involved in focus groups across LMIC sites, nor do they speak their languages, hence they might miss understand behavior, perceptions, and cultural nuances from other sites of the study. To address this limitation, the researchers reached out to local stakeholders to gain clarifications on some parts in the translated transcripts that weren’t clear or seemed to use local colloquialisms. Additional contextual information referring to local implementation characteristics was collected from each local site to increase the trustworthiness of the interpretation process (see supplementary material 1). The final analysis was also presented to site authors who were invited to critically comment on the manuscript. While it may be impossible to fully grasp cultural nuances and local processes, these different strategies assured that central behaviors, perceptions and cultural context  were clarified, verified, and gauged  [50]. Finally, this study lacked the ability to address gender issues, because only 2 sites delivered the intervention in gender segregation. Future studies should be mindful of gender aspects which may have implications to experiences of PSWs.

## Supplementary Information

Below is the link to the electronic supplementary material.

Supplementary file1 (DOCX 50 KB)

## Data Availability

No datasets were generated or analyzed during the current study.
