# Supplementary material for: Multicultural Implementation-Experiences of Peer Support Workers in MH Services: Qualitative Findings of UPSIDES Innovative Intervention—An International Multi-Site Project
Source: J Occup Rehabil. 2025 Nov 3;36(3):863–76. doi: 10.1007/s10926-025-10320-4 (PMC13364822; doi:10.1007/s10926-025-10320-4)
Supplement: Supplementary file 1 — Supplementary file1 (DOCX 50 KB) [file 10926_2025_10320_MOESM1_ESM.docx]

**Supplementary material 1: Context of the study sites**

| **City, Country** | Ulm/  Guenzburg, Germany  (Ulm) | Hamburg,  Germany  (UKE) | Butabika, Uganda  (BU) | Dar es Salaam, Tanzania  (DS) | Be’er Sheva,  Israel  (BGU) | Pune, India  (PU) |
| --- | --- | --- | --- | --- | --- | --- |
| **Income level** | High-income | High-income | Low-income | Low-income | High-income | Lower-middle-income |
| **Implementation at/in** ^[1]^ | Catchment area of Ulm  University’s Department of Psychiatry and  Psychotherapy II; District Hospital Ulm/ Guenzburg | Catchment area of University Medical Centre Hamburg-Eppendorf and  community  services | Butabika Hospital, Kampala | Muhimbili  National Hospital at the Department of Psychiatry  and Mental Health | Kidum Proyektim Shikumim (supported residential and mentoring services); Enosh (Israeli Mental Health Association) | Hospital for Mental  Health in Ahmedabad, Gujarat |
| **PSW involved prior to UPSIDES**  ^[1]^ | No/little | Yes | Yes | No | Yes | Yes |
| **MH services in which UPSIDES PSWs are involved in*** | Outpatient  services | Outpatient services | In- and outpatient  services | In- and outpatient  services | Outpatient  services | Inpatient services |
| **Area of UPSIDES peer support*** | Rural area | Urban area | Mixed area | Urban area | Urban area | Urban area |

*Information provided by research workers at study sites

**References**

1. Moran GS, Kalha J, Mueller-Stierlin AS, Kilian R, Krumm S, Slade M, et al. Peer support for people with severe mental illness versus usual care in high-, middle- and low-income countries: study protocol for a pragmatic, multicentre, randomised controlled trial (UPSIDES-RCT). Trials. 2020;21:371. doi:10.1186/s13063-020-4177-7.

**Supplementary File 2: Topic Guide**

| Title | Instructions WP6 focus group #2 for UPSIDES Peer Support Workers |
| --- | --- |
| WP No | WP 6 |
| Task lead | BGU, UUlm |

The purpose of this guideline is to facilitate the focus group discussion with PSWs.

**How to use the topic guide:** The topic guide includes five topics, which are to be discussed during the focus group session. For each topic there is a main question (written in the grey field). Please ask the main question for every topic. In addition to the main questions, there are several additional questions, which should be asked if themes are not emerging during the discussion of the topic. Try to allow themes to come up naturally but prompt the PSWs with the additional questions as necessary. Ensure that all the sub-topics written in the left-hand column of each topic are addressed in the discussion.

**Style of interviewing**: use non-judgmental probing to help participants elaborate on answers; use open questions to introduce a new topic; use clarifying questions to probe a topic; at the end of the interview thank the participants for their contribution. Kindly note that the interviewer is in the role of a researcher and not in the role as a mental health professional.

Interviewer ID ___ ___ ___ ___

Interview date (DD/MM/YYYY): __ __ / __ __ / __ __ __

Location of the interview __________________________________

Country: _________________________________

Ensure that the participants are comfortable.

PLEASE TRANSLATE FROM HERE

**Introduction**

Say:

- *Hi and welcome to this meeting. My name is ___ and my colleague is___. Thank you for taking the time to talk to us. As you know, in the UPSIDES study we are examining many aspects of peer support work. Now that you have delivered UPSIDES peer support intervention, we have invited you today to discuss your experience of being an UPSIDE peer support worker. This includes your perspectives of being trained and working in this project, and any benefits or challenges you believe you and your peers (service users) have experienced as a result of the intervention.*
- *What we discuss here today remains confidential. We ask you not to share any personal information that is discussed here with anyone outside this group. We store the data safely so that no one outside the research team has access to it. We will assign a number to you so we will never use your name or any other identifiable information.*
- *Your participation in this study is voluntary, and you can withdraw at any time and this will not affect your benefits or medical care whatsoever.*
- *The interview will be recorded using an audio recorder so that we don’t miss any of your useful comments. Feel free to discuss with each other. There are no right or wrong answers. You are the experts who we would like to learn from.*
- *The discussion will take around 60 minutes, depending also on how much you want to share. I am the moderator, and I will guide the discussion today. My colleague will take notes of the discussion so that we will not miss any of your valuable contribution in case something goes wrong with the recorder, and also so that we can note who said what when we transcribe the discussion later.*
- *Before we start the discussion, I would like you to read carefully the information and sign the consent form if you would like to proceed with the discussion.*

**Hand out participant information and informed consent** (attached above)

- *Before we begin, I would like to know if you have any questions.*

Explain you are starting the audio recorder now.

Time interview started: __________________

**Topic 1: PSWs Experiences in training and working in UPSIDES**

Main Question: What is **your overall experience of receiving UPSIDES training and providing UPSIDES peer support?**

| Training to become an UPSIDES PSW | Please describe the experience of training to become an UPSIDES PSW?) what worked well? What did not go well?)  Would you add anything/change anything in the training? |
| --- | --- |
| UPSIDES intervention experience | Please describe the experience of working as a PSW in the UPSIDES project (what worked well? What did not go well?)  Would you add anything/change anything in the intervention?  What is core to this role? (Please give examples from your work as an UPSIDES PSW) |
| Using lived experience | UPSIDES PSWs use their lived experience with service users. What are your views about using lived experience as PSWs?  What works well? What does not? (Ask for examples from their UPSIDES PSW intervention) |
| Vocational development | How did UPSIDES peer support project impact your individual (personal) occupational development? (Gaining skills, knowledge, career, personal development) |

For maintaining conversation: *Can you describe this more detailed? Can you give an example?*

**Topic 2: UPSIDES effects on service users**

**Main Question: What are the main** effects of UPSIDES on service users?

| UPSIDES optimal effects | From your experiences, what was the most significant contribution of UPSIDES peer support to service users? (Give examples)  What is additional positive impact of UPSIDES on service users? (Give examples) |
| --- | --- |
| UPSIDES negative effects | Were there negative effects of UPSIDES for some of the service users? If yes – please describe and explain why you think they occurred?  What was most challenging about UPSIDES for service users? (Give examples) |

*Now we move to talk about organizational issues related to UPSIDES*

**Topic 3: Organizational issues (contextual factors, barriers, and facilitators)**

**Main Question: What are the** barriers, facilitators and needs for the success of UPSIDES in your mental health setting?

| Overall impression of impact | As an UPSIDES PSW, what is your general impression of the institution in which you conducted UPSIDES intervention? (Was it open to accept UPSIDES PSW?)  What was the impact of UPSIDES on the institution? |
| --- | --- |
| Organizational factors & resources contributing to UPSIDES | Were there any factors/resources in the institution that helped practice UPSIDES peer support? (Facilities, accessibility, role clarity, peer network, training, team culture?) |
| Barriers and challenges | What made it difficult to conduct UPSIDES peer support in your institution? (Work culture, organizational structure, budget, other things) |
| Improvements | From your experience, what are some recommendations you would give for training and organizations to improve the UPSIDES practice of peer support? |

**Topic 4: COVID-19**

**Main Question: Please describe if and how COVID-19 has affected you and your work in UPSIDES as a PSW?**

| Description & details | Please describe any changes and influences that COVID-19 posed to UPSIDES |
| --- | --- |
| Managing Covid related changes | How did you manage some of these changes? (Creative solutions? New ways of connecting and how?) |
| PSW UPSIDES contribution to COVID-19 situation | -Was there a specific advantage to being an UPSIDES PSW during COVID-19?  -Was there a specific advantage UPSIDES PSW intervention for service users during COVID-19?  -What else could help overcome COVID-19 challenges? |

**Topic 5: Anything else**

**Main Question: Is there anything else you would like to add?**

Please wait and leave some space for participants to think and talk.

**End**

- Say:

*Thank you for your time. We will treat the information you gave to us today confidentially. We kindly ask you do not talk to people outside the group about what was said in the group*

- Time interview end: ________________

Interviewer comments on how the interview went e.g., key themes, important insights, emotional tone. Please fill out immediately after the focus group:

**Supplementary file 3:**

**Consolidated criteria for reporting qualitative studies (COREQ): 32-item checklist**

**Domain 1: Research team and reflexivity**

*Personal Characteristics*

1. Interviewer/facilitator: Which author/s conducted the interview or focus group? Two research workers from every site facilitated the FGDs (in total 12 persons).

2. Credentials: What were the researcher’s credentials? E.g. PhD, MD Research workers had mixed backgrounds, including study degrees (bachelor, master).

3. Occupation: What was their occupation at the time of the study? FGD facilitators were research workers in the UPSIDES project.

4. Gender: Was the researcher male or female? Most of the researchers were female, some male.

5. Experience and training: What experience or training did the researcher have? Online training sessions for interviewers to introduce the topic guides were conducted, potential challenges during the FGDs were discussed. The researchers who directly analysed the data had several years of experience with qualitative research.

*Relationship with participants*

6. Relationship established: Was a relationship established prior to study commencement? Yes, in in most study sites interviewer were known as part of the UPSIDES project before the start of the FGDs.

7. Participant knowledge of the interviewer: What did the participants know about the researcher? e.g. personal goals, reasons for doing the research. Participants had mixed knowledge on the interviewers, depending on whether or not they knew the interviewers and the project beforehand. In all study sites the interviewers introduced themselves, the study and the study aim before the start of the FGD.

8. Interviewer characteristics: What characteristics were reported about the interviewer/facilitator? e.g. Bias, assumptions, reasons and interests in the research topic. Personal characteristics about the interviewers were not reported.

**Domain 2: study design**

*Theoretical framework*

9. Methodological orientation and Theory: What methodological orientation was stated to underpin the study? e.g. grounded theory, discourse analysis, ethnography, phenomenology, content analysis. Transcripts were analysed using qualitative thematic analysis and MAXQDA software.

*Participant selection*

10. Sampling: How were participants selected? e.g. purposive, convenience, consecutive, snowball. A purposive sample strategy was used.

11. Method of approach: How were participants approached? e.g. face-to-face, telephone, mail, email. Participants were contacted face-to-face, telephone and email.

12. Sample size: How many participants were in the study? 38.

13. Non-participation: How many people refused to participate or dropped out? Reasons? Several participants dropped out due time constraints.

*Setting*

14. Setting of data collection: Where was the data collected? e.g. home, clinic, workplace. FGDs were mainly conducted in the organisations where UPSIDES was implemented (e.g. hospital, community service).

15. Presence of non-participants: Was anyone else present besides the participants and researchers? No.

16. Description of sample: What are the important characteristics of the sample? e.g. demographic data, date. Please see table 1 and appendix of the context of the study sites.

*Data collection*

17. Interview guide: Were questions, prompts, guides provided by the authors? Was it pilot tested? Semi-structured topic guide were used which were harmonised across sites. Each site interviewer followed thes guidelines and could add questions of their own if they felt the group was discussing additional relevant topics.

18. Repeat interviews: Were repeat interviews carried out? If yes, how many? No.

19. Audio/visual recording: Did the research use audio or visual recording to collect the data? Audio recording was used.

20. Field notes: Were field notes made during and/or after the interview or focus group? Yes.

21. Duration: What was the duration of the interviews or focus group? 32 to 130 minutes.

22. Data saturation: Was data saturation discussed? Yes, we conducted 9 focus groups across all research sites. These FG involved the majority of PSWs who delivered the intervention which allowed sufficient saturation.

23. Transcripts returned: Were transcripts returned to participants for comment and/or correction? No.

**Domain 3: analysis and findings**

*Data analysis*

24. Number of data coders: How many data coders coded the data? Three

25. Description of the coding tree: Did authors provide a description of the coding tree? Authors provided a description of the thematic topics identified.

26. Derivation of themes: Were themes identified in advance or derived from the data? Both ways.

27. Software: What software, if applicable, was used to manage the data? MAXQDA 2020

28. Participant checking: Did participants provide feedback on the findings? No

*Reporting*

29. Quotations presented: Were participant quotations presented to illustrate the themes / findings? All themes are supported by quotes resulting from the MAXQDA analytic procedure and researcher’s analyses. Demonstrative quotations can be found in the result section. Was each quotation identified? e.g. participant number. Yes

30. Data and findings consistent: Was there consistency between the data presented and the findings? Yes

31. Clarity of major themes: Were major themes clearly presented in the findings? Yes

32. Clarity of minor themes: Is there a description of diverse cases or discussion of minor themes? More themes that related to findings already well known, such as more nuanced benefits for recovery were mentioned but not expanded on in this study which focused on the most innovative findings to PSWs experiences of implementation of a peer intervention.
